# Apelin Promotes Endothelial Progenitor Cell Angiogenesis in Rheumatoid Arthritis Disease *via* the miR-525-5p/Angiopoietin-1 Pathway

**DOI:** 10.3389/fimmu.2021.737990

**Published:** 2021-09-29

**Authors:** Ting-Kuo Chang, You-Han Zhong, Shan-Chi Liu, Chien-Chung Huang, Chun-Hao Tsai, Hsiang-Ping Lee, Shih-Wei Wang, Chin-Jung Hsu, Chih-Hsin Tang

**Affiliations:** ^1^ Department of Medicine, Mackay Medical College, New Taipei, Taiwan; ^2^ Division of Spine Surgery, Department of Orthopedic Surgery, MacKay Memorial Hospital, New Taipei, Taiwan; ^3^ Graduate Institute of Biomedical Science, China Medical University, Taichung, Taiwan; ^4^ Department of Medical Education and Research, China Medical University Beigang Hospital, Yunlin, Taiwan; ^5^ School of Medicine, China Medical University, Taichung, Taiwan; ^6^ Division of Immunology and Rheumatology, Department of Internal Medicine, China Medical University Hospital, Taichung, Taiwan; ^7^ Department of Sports Medicine, College of Health Care, China Medical University, Taichung, Taiwan; ^8^ Department of Orthopedic Surgery, China Medical University Hospital, Taichung, Taiwan; ^9^ School of Chinese Medicine, China Medical University, Taichung, Taiwan; ^10^ Department of Chinese Medicine, China Medical University Hospital, Taichung, Taiwan; ^11^ Department of Medicine, MacKay Medical College, New Taipei City, Taiwan; ^12^ Graduate Institute of Natural Products, College of Pharmacy, Kaohsiung Medical University, Kaohsiung, Taiwan; ^13^ Institute of Biomedical Sciences, Mackay Medical College, Taipei, Taiwan; ^14^ Chinese Medicine Research Center, China Medical University, Taichung, Taiwan; ^15^ Department of Biotechnology, College of Health Science, Asia University, Taichung, Taiwan

**Keywords:** apelin, angiopoietin-1, endothelial progenitor cells (EPC), rheumatoid arthritis, miR-525-5p

## Abstract

Angiogenesis is a critical process in the formation of new capillaries and a key participant in rheumatoid arthritis (RA) pathogenesis. The adipokine apelin (APLN) plays critical roles in several cellular functions, including angiogenesis. We report that APLN treatment of RA synovial fibroblasts (RASFs) increased angiopoietin-1 (Ang1) expression. Ang1 antibody abolished endothelial progenitor cell (EPC) tube formation and migration in conditioned medium from APLN-treated RASFs. We also found significantly higher levels of APLN and Ang1 expression in synovial fluid from RA patients compared with those with osteoarthritis. APLN facilitated Ang1-dependent EPC angiogenesis by inhibiting miR-525-5p synthesis *via* phospholipase C gamma (PLCγ) and protein kinase C alpha (PKCα) signaling. Importantly, infection with APLN shRNA mitigated EPC angiogenesis, articular swelling, and cartilage erosion in ankle joints of mice with collagen-induced arthritis. APLN is therefore a novel therapeutic target for RA.

## Introduction

Rheumatoid arthritis (RA) is one of the most common autoimmune disorders, characterized by the accumulation of inflammatory cytokines in the synovial joint, resulting in pannus formation, cartilage degradation and bone destruction (1). Angiogenesis is a critical driver of RA disease, in which pre-existing blood vessels promote the entry of blood-derived leukocytes into the synovial tissues to facilitate and potentiate inflammation (2).

Endothelial progenitor cells (EPCs) develop from bone marrow-derived endothelial stem cells, which contain the cell surface markers CD133, CD34 and vascular endothelial growth factor receptor 2 (VEGFR2) and are capable of stimulating postnatal vasculogenesis (3) and angiogenic function (4). EPC proliferation and migration facilitate angiogenesis (4), enabling the development of RA (5, 6). EPC-dependent angiogenesis therefore seems to be a worthwhile treatment target in RA. EPC proliferation, migration and angiogenesis is regulated by the balance in activities between proangiogenic factors such as vascular endothelial growth factor (VEGF), platelet-derived growth factor (PDGF) and angiopoietin-1 (Ang1), and antiangiogenic factors including thrombospondin-1 (7, 8). Ang1 plays a critical role in endothelial cell adhesion, migration and production during angiogenesis (9). However, the effects of Ang1 in EPC angiogenesis in RA disease are unclear.

Apelin (APLN) is a member of the adipokine superfamily that is expressed in different human tissues including nervous system, adipose and endothelial tissues (10, 11). APLN has been linked with numerous disorders, including cardiovascular and neurodegenerative diseases (10, 11). Emerging evidence has highlighted the association between APLN and arthritic diseases, including RA and osteoarthritis (OA), for example (12). Treating human chondrocytes with APLN increases the synthesis of matrix metalloproteinases (MMPs) and other catabolic factors (13). APLN also promotes the production of the proinflammatory cytokine interleukin 1 beta (IL-1β) in human OA synovial fibroblasts (OASFs) (14). In RA patients, levels of APLN expression are associated with the expression of the catabolic enzyme MMP-9 (15). These reports suggest that APLN is a novel avenue for treating arthritic diseases.

MiRNAs are single-stranded noncoding RNA molecules that manipulate gene expression at the post-transcriptional level (16). Various miRNA genes expressed in immune, inflammatory and synovial cells from patients with RA (17) can cause synovial hyperplasia and bone damage, or promote inflammation, through positive or negative manipulation (18). Recently, miRNAs have been found to regulate angiogenic activity in the progression of arthritic diseases (19, 20). However, it remains unclear as to how the APLN-miRNA axis regulates angiogenesis in RA disease. Our study has identified higher levels of APLN and Ang1 expression in patients with RA than in those with OA. APLN treatment increased RASF-derived Ang1 production and facilitated EPC angiogenesis by inhibiting miR-525-5p synthesis *via* phospholipase C gamma (PLCγ) and protein kinase C alpha (PKCα) signaling. Inhibition of APLN expression diminished Ang1-dependent angiogenesis and inhibited collagen-induced arthritis (CIA) in mice. APLN is therefore a novel therapeutic target for RA.

## Materials and Methods

### Materials

APN, Ang1, PLCγ and PKCα antibodies were purchased from Santa Cruz Biotechnology (CA, USA). p-PLCγ and p-PKCα antibodies were purchased from Cell Signaling Technology (Danvers, MA, USA). All siRNAs (ON-TARGET*plus*) were obtained from Dharmacon Research (Lafayette, CO, USA). Taqman^®^ one-step PCR Master Mix, qPCR primers and probes were obtained from Applied Biosystems (Foster City, CA, USA). β-Actin antibody and pharmacological inhibitors were obtained from Sigma-Aldrich (St. Louis, MO, USA).

### Human Synovial Fluid Samples

Study approval was granted by the Institutional Review Board of China Medical University Hospital (Taichung, Taiwan) and all patients provided written informed consent before participating in the study. Synovial fluid samples were obtained from patients undergoing total knee arthroplasty for OA (n=20) or RA (n=20).

### Cell Culture

Human RASFs were purchased from the Riken Cell Bank (Ibaraki, Japan). Primary human EPCs were isolated according to the procedure detailed in our previous reports (21, 22). Mouse osteoblastic cell line MC3T3-E1 were purchased from the American Type Culture Collection (Manassas, VA, USA). RASFs and EPCs were maintained in DMEM while MC3T3-E1 cells were cultured in α-MEM medium. The culture mediums were supplemented with 20 mM HEPES and 10% fetal bovine serum, 2 mM glutamine, penicillin (100 U/ml) and streptomycin (100 μg/ml) at 37°C with 5% CO_2_.

### Western Blot Analysis

RASF cells (5 × 10^5^ cells) were seeding in 6 well plate. Cell lysates were resolved by SDS-PAGE and transferred to Immobilon^®^ PVDF membranes. Western blot analysis was performed according to the procedures detailed in our previous investigations (23–26).

### Quantitative Real-Time PCR (qPCR) Analysis of mRNA and miRNA

Total RNA was extracted from RASFs and paws using TRIzol reagent and then reverse-transcribed into cDNA using oligo(dT) primers. For the miRNA assay, cDNA was synthesized using the TaqMan MicroRNA Reverse Transcription Kit. qPCR analysis was conducted according to an established protocol (27–29).

### Preparation of Conditioned Medium (CM)

RASFs were plated in 6-well dishes and grown to confluence. The culture medium was exchanged with serum-free DMEM medium. CM were collected 1 days after the change of media and stored at -20°C until use. In the series of experiments, cells were pretreated for 30 min with inhibitors, including U73122, GF109203X and Go6976 or transfected with miR-525-5p mimic, PLCγ, PKCα and PKCδ siRNA for 24 h followed by treatment with APLN for 24 h to prevent signaling *via* the APLN pathway.

### ELISA Assay

RASFs were treated with pharmacological inhibitors then incubated with APLN for 24 h and the medium was quantified for Ang1 expression using a Ang1 ELISA kit (Peprotech, Rocky Hill, NJ, USA), following the manufacturer’s protocol.

### EPC Migration and *In Vitro* Tube Formation

EPCs were treated with RASF conditioned medium (CM) for 24 h. EPC migration and *in vitro* tube formation were evaluated by the procedures detailed in our previous publication (30).

### The Chick Chorioallantoic Membrane Assay

*In vivo* angiogenic activity was assessed using the chorioallantoic membrane (CAM) of the chick embryo, as described previously (6, 31). Fertilized chick embryos were incubated in an 80% humidified atmosphere at 37°C. All animal investigations adhered to approved protocols issued by the Institutional Animal Care and Use Committee of China Medical University (Taichung, Taiwan).

### *In Vivo* Matrigel Plug Assay

Four-week-old nude male mice received a single subcutaneous injection of Matrigel containing RASF CM. Mice were subcutaneously injected with 300 μL of Matrigel. On day 7, the Matrigel plugs were harvested, partially fixed with 4% formalin, embedded in paraffin, and subsequently processed for immunohistochemistry staining for CD31, CD34, and CD133. Hemoglobin concentrations were measured, according to previously described methodology (6, 31, 32).

### CIA Mouse Model

The CIA mouse model was performed according to the methodology detailed in our previous publications (6, 32, 33). After receiving two immunizations, the mice were given weekly intra-articular injections of ~7.1 × 10^6^ plaque-forming units (PFU) of control or APLN short hairpin RNA (shRNA). Upon sacrifice after 49 days of treatment, phalanges and ankle joints were removed from each mouse then fixed in 4% paraformaldehyde for micro-computed tomography (micro-CT) analysis. Analysis was performed using CTAn 1.18.4 (Bruker micro-CT, Kontich, Belgium). First, we segmented the reaction area which showed less calcium content with porous structure. We then labelled the isolated reaction area with purple color.

### Statistical Analysis

All statistical analyses were carried out using GraphPad Prism 5.0 (GraphPad Software) and all values are expressed as the mean ± S.D. Differences between selected pairs from the experimental groups were analyzed for statistical significance using the paired sample *t*-test for *in vitro* analyses and by one-way ANOVA followed by Bonferroni testing for *in vivo* analyses. * *p* < 0.05, ** *p* < 0.01 and *** *p* < 0.001 *versus* the control group; # *p* < 0.05 *versus* the APLN-treated group.

## Results

### APLN Facilitates Ang1-Dependent EPC Angiogenesis

**Figure 1:**
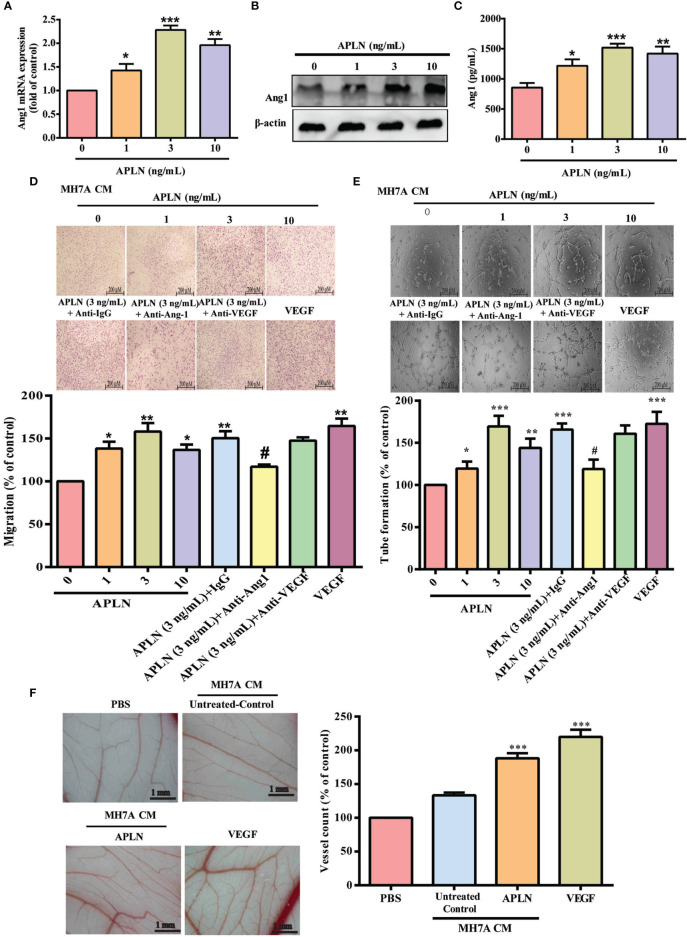
APLN increases Ang1 expression in RASFs and promotes EPCs tube formation and migration. **(A–C)** RASFs were incubated for 24 h with APLN; Ang1 expression was quantified by qPCR, Western blot and ELISA assays. **(D, E)** RASFs cells were treat with APLN does manner for 24 h and collected conditioned medium (CM) was applied to endothelial progenitor cells (EPCs), then EPC migration and angiogenesis was measured. **(F)** Matrigel plugs containing CM from APLN-treated RASFs or VEGF (positive control) were applied to 6-day-old fertilized chick embryos for 4 days. CAMs were examined by microscopy and photographed, and vessels counted. *p < 0.05, **p < 0.01 and ***p < 0.001 *versus* the control group; ^#^ *p* < 0.05 *versus* the APLN-treated group.

APLN is associated with the progression of arthritic diseases, including RA (12). Ang1 is an important angiogenetic regulator in endothelial cell angiogenesis (9). First, we examined whether APLN promotes Ang1 synthesis in RASFs. Stimulation of RASFs with APLN dose-dependently increased Ang1 transcription and translation levels (**Figures 1A, B**) and also the secretion of Ang1 protein (**Figure 1C**). EPC tube formation and migration assays examined the effects of APLN-controlled angiogenesis in RASFs (5). CM from APLN-treated RASFs significantly increased the formation and reorganization of capillary-like network structures as well as migratory activity (VEGF-increased vessel formation served as the positive control) (**Figures 1D, E**). Treatment with Ang1 but not VEGF antibody, dramatically reduced the effects of CM from APLN-treated RASFs upon EPC tube formation and migration (**Figures 1D, E**), indicating that Ang1 is more important than VEGF in APLN-promoted EPC angiogenesis. To directly examine whether APLN acts as an angiogenic factor *in vivo*, the CAM assay was used. Matrigel was mixed with CM from APLN-treated RASFs and placed onto the surface of the CAMs. CM from APLN-treated RASFs synthesized new capillaries then control (VEGF-increased vessel formation served as the positive control) (**Figure 1F**), suggesting that APLN promotes Ang1 production in RASFs and enhances tube formation and migration of EPCs.

### High Levels of APLN and Ang1 Expression in RA Patients Induce EPC Homing and Angiogenesis

Next, we investigated APLN and Ang1 levels in RA patients. We found markedly higher levels of APLN and Ang1 expression in synovial fluid from RA patients compared with OA synovial fluid samples (**Supplementary Figures 1A, B**). Synovial fluid levels of APLN and Ang1 were positively correlated (**Supplementary Figure 1C**). Next, we examined whether synovial fluid from RA patients promotes EPC homing and angiogenesis. Migratory activity, as well as the formation and reorganization of capillary-like network structures, was significantly greater in EPCs incubated with RA synovial fluid compared with EPCs incubated with OA synovial fluid (**Supplementary Figures 1D, E**). Treatment with Ang1 antibody dramatically diminished the effects of RA synovial fluid upon EPC migration and tube formation (**Supplementary Figures 1D, E**), indicating that high levels of APLN and Ang1 expression in RA patients induce EPC homing and angiogenesis.

### PLCγ and PKCα Signaling Cascades Regulate APLN-Promoted Ang1 Expression and Angiogenesis in EPCs

**Figure 2:**
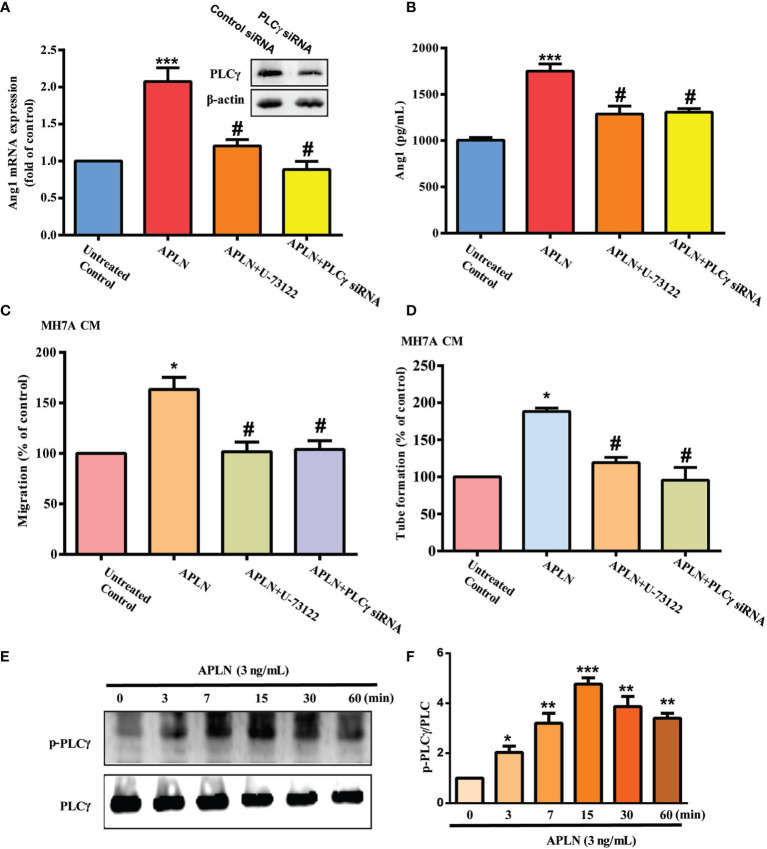
PLCγ signaling regulates APLN-induced effects on Ang1 expression and EPC angiogenesis. **(A, B)** RASFs were left untreated, stimulated with APLN (3 ng/mL) alone for 24 h, or pretreated with U73122 for 30 min, or transfected with PLCγ siRNA for 30 min, prior to 24 h of APLN (3 ng/mL) stimulation. Ang1 expression was examined by qPCR and ELISA assays. **(C, D)** Collected CM was applied to EPCs, and angiogenesis was determined. **(E)** RASFs were treated with APLN for varying amounts of time and Western blot (n=3) determined PLCγ phosphorylation. **(F)** Densitometry analysis of **(E)**. *p < 0.05, **p < 0.01 and ***p < 0.001 *versus* the control group; ^#^ *p* < 0.05 *versus* the APLN-treated group.

**Figure 3:**
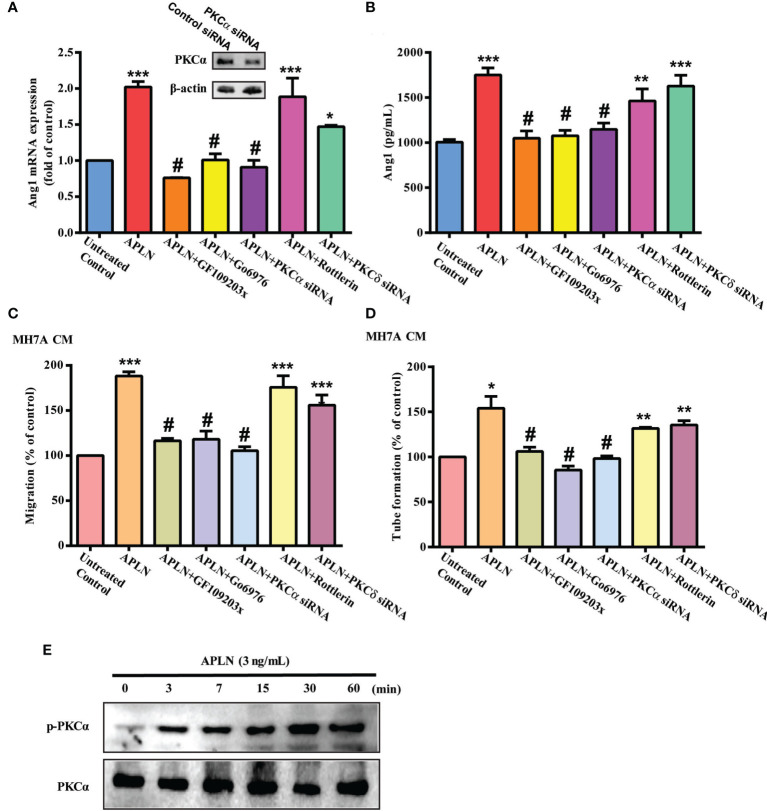
PKCα signaling regulates APLN-induced effects on Ang1 expression and EPC angiogenesis. **(A, B)** RASFs were pretreated with GF109203X and Go6976 for 30 min, or transfected with PKCα and PKCδ siRNAs for 24 h, then stimulated with APLN for 24 h. Ang1 expression was examined by qPCR and ELISA assays. **(C, D)** Collected CM was applied to EPCs, and EPC angiogenesis was determined. **(E)** RASFs were treated with APLN time-manner and Western blot (n=3) determined PKCα phosphorylation. *p < 0.05, **p < 0.01 and ***p < 0.001 *versus* the control group; ^#^ *p* < 0.05 *versus* the APLN-treated group.

PLC and PKC signaling pathways control different cellular functions, including angiogenesis (34). We therefore sought to determine how these pathways affect APLN-induced upregulation of Ang1 synthesis and EPC angiogenesis. Treatment of RASFs with a PLC inhibitor (U73122) or PLCγ siRNA reduced the effects of APLN upon Ang1 expression (**Figures 2A, B**) and inhibited APLN-induced upregulation of EPC migration and tube formation (**Figures 2C, D**). Incubating RASFs with APLN induced PLCγ phosphorylation (**Figures 2E, F**). In addition, the PKCα inhibitors (GF109203X and Go6976) and PKCα siRNA, but not the PKCδ inhibitor (Rottlerin) or PKCδ siRNA, abolished APLN-facilitated Ang1 expression and EPC angiogenesis (**Figures 3A–D**). Stimulation of RASFs with APLN also increased PKCα phosphorylation (**Figure 3E**). Thus, APLN promotes Ang1 expression in RASFs and enhances EPC angiogenesis *via* PLCγ and PKCα signaling.

### Inhibition of miR-525-5p Controls APLN-Promoted Ang1 Synthesis and EPC Angiogenesis

**Figure 4:**
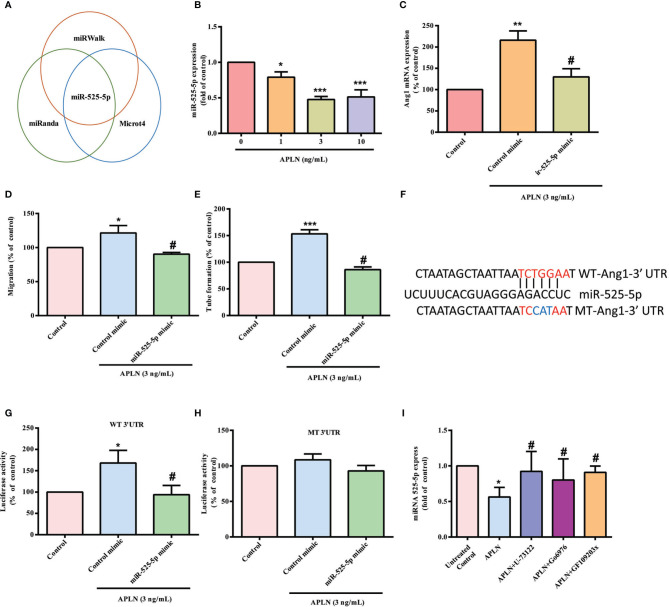
APLN facilitates Ang1 synthesis and EPC angiogenesis by inhibiting miR-525-5p. **(A)** Open-source software enabled identification of miRNAs that possibly interfere with Ang1 transcription. **(B)** RASFs were incubated with APLN for 24 h. miR-525-5p expression was determined by the qPCR assay. **(C)** RASFs were transfected with miR-525-5p mimic for 24 h, then stimulated with APLN for 24 h. Ang1 levels were determined by qPCR. **(D, E)** Collected CM was applied to EPCs, and EPC angiogenesis was quantified. **(F)** Schematic 3′UTR representation of human Ang1 containing the miR-525-5p binding site. **(G, H)** RASFs were transfected with the indicated luciferase plasmid with or without miR-525-5p mimic for 24 h, then stimulated with APLN for 24 h. Relative luciferase activity was determined. **(I)** RASFs were pretreated with U73122, GF109203X and Go6976 for 30 min, then stimulated with APLN for 24 h. miR-525-5p expression was quantified by qPCR. *p < 0.05, **p < 0.01 and ***p < 0.001 *versus* the control group; ^#^ *p* < 0.05 *versus* the APLN-treated group.

The dysregulated expression of miRNAs in patients with RA differs from miRNA expression in healthy individuals (35, 36). Using open-source miRNA software (miRanda, https://bioweb.pasteur.fr/packages/pack@miRanda@3.3a/; Microt4, http://diana.imis.athena-innovation.gr/DianaTools/index.php?r=microtv4/index; and miRWalk, http://mirwalk.umm.uni-heidelberg.de/), we identified 8 candidate miRNAs that could possibly bind to the 3’UTR region of Ang1 mRNA. Among these 8 miRNAs, levels of miR-525-5p expression were suppressed by the greatest extent after APLN administration (**Figure 4A** and **Supplementary Figure S2**). Treating RASFs with APLN concentration-dependently reduced miR-525-5p synthesis (**Figure 4B**). Transfection of RASFs with miR-525-5p mimic antagonized the effects of APLN upon Ang1 production and EPC angiogenesis (**Figures 4C–E**). Similarly, transfection with miR-525-5p mimic downregulated Ang-1 expression in mouse MC3T3-E1 cells (**Supplementary Figure S3**).

To examine whether miR-525-5p regulates *ANG1* gene transcription, we constructed a luciferase reporter vector with the wild-type 3’UTR of *ANG1* mRNA (wt-Ang1-3’UTR) and a mutated vector harboring mismatches in the predicted miR-525-5p binding site (mt-Ang1-3’UTR) (**Figure 4F**). MiR-525-5p mimic reduced APLN-induced luciferase activity in the wt-Ang1-3’UTR plasmid, but not in the mt-Ang1-3’UTR plasmid (**Figures 4G, H**). Moreover, U73122, GF109203X and Go6976 all reversed APLN-induced inhibition of miR-525-5p expression (**Figure 4I**), indicating that PLCγ and PKCα signaling mediate APLN-induced inhibition of miR-525-5p.

### Inhibition of APLN Reduces EPC Angiogenesis as Well as Arthritis Severity *In Vivo*

**Figure 5:**
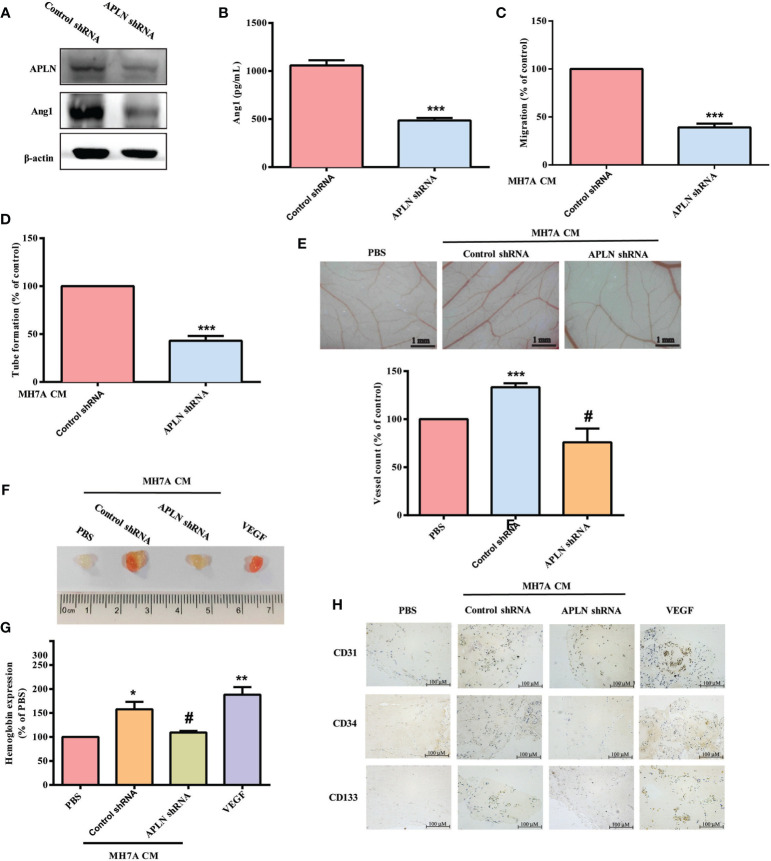
Inhibition of APLN reduces EPC angiogenesis *in vivo*. **(A, B)** RASFs were infected with APLN shRNA for 24 h. APLN and Ang1 expression was examined by Western blot (n=3) and ELISA. **(C, D)** Collected CM was applied to EPCs, and EPC angiogenesis was quantified. **(E)** After subjecting RASFs to the treatment conditions as indicated, the harvested CM was applied to 6-day-old fertilized chick embryos for 4 days. CAMs were examined by microscopy and photographed, and vessels were counted manually. **(F, G)** Matrigel plugs containing the harvested CM were subcutaneously injected into the flanks of nude mice. After 7 days, the plugs were photographed, and hemoglobin levels were quantified. **(H)** Plug specimens were immunostained with CD31, CD34 and CD133 antibodies. *p < 0.05, **p < 0.01 and ***p < 0.001 *versus* the control group; ^#^ *p* < 0.05 *versus* the APLN-treated group.

APLN shRNA was used to validate the *in vivo* role of APLN. Infection of RASFs with APLN shRNA reduced APLN and Ang1 levels (**Figures 5A, B**). Compared with RASF CM, APLN shRNA-infected RASF CM reduced EPC migration and tube formation (**Figures 5C, D**). CAM and Matrigel investigations demonstrated that CM from RASFs enhanced vessel formation *in vivo* (**Figures 5E, F**), while APLN shRNA reduced RASF CM-promoted induction of vessel formation (**Figures 5E, F**). These results were confirmed by IHC staining of hemoglobin levels and the human-specific vessel marker CD31, as well as EPC markers CD34 and CD133 (**Figures 5G, H**).

**Figure 6:**
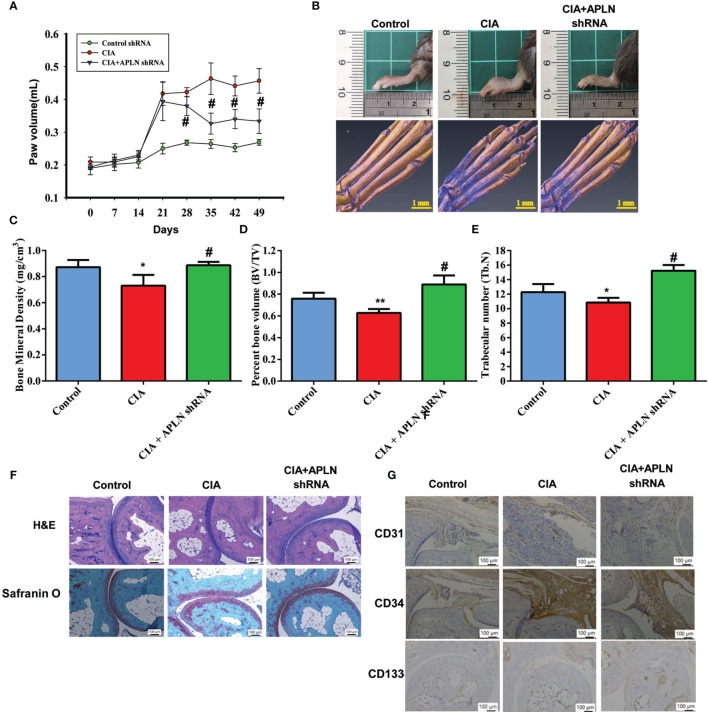
APLN knockdown reduces angiogenesis and the severity of RA *in vivo*. CIA mice received intra-articular injections of 7.1 × 10^6^ PFU APLN shRNA or control shRNA on day 14 and were euthanized on day 49. **(A)** In comparisons of digital plethysmometer values for the amounts of hind paw swelling in the CIA and APLN shRNA groups, the statistical comparisons were not significant on days 14 and 21 (p=0.5 and p=0.3, respectively), but they were significant change on days 14, 21, 28,35,42 and 49 (p=0.001, p<0.0001, p<0.0001, and p<0.0001, respectively). **(B)** Representative micro-CT images of the hind paws taken on day 49. **(C–E)** Micro-CT SkyScan Software quantified bone mineral density, bone volume and trabecular numbers. **(F, G)** Histological sections of ankle joints were stained with H&E or Safranin O and immunostained with CD31, CD34 and CD133. *p < 0.05 versus the control group; ^#^ *p* < 0.05 *versus* the APLN-treated group. *p < 0.05 and **p < 0.01 *versus* the control group; ^#^ *p* < 0.05 *versus* the CIA group.

Next, we used the CIA mouse model to investigate the therapeutic effect of inhibiting APLN *in vivo*. Compared with controls, CIA mice exhibited significant paw swelling that improved after administration of APLN shRNA (**Figures 6A, B**). Micro-CT images of the hind paws showed that APLN shRNA reversed CIA-induced reductions in bone mineral density (p<0.01), bone volume (p<0.0001) and trabecular numbers (p<0.0001) (**Figures 6C–E**). Moreover, CIA mice exhibited lower cartilage thicknesses, as indicated by H&E and Safranin-O/Fast-green staining (**Figure 6F**). APLN shRNA reversed CIA-induced cartilage degradation (**Figure 6G**). According to IHC staining data, levels of vessel marker (CD31) and EPC markers (CD34 and CD133) were all markedly higher in CIA mice than in controls. Notably, APLN shRNA treatment antagonized CIA-induced upregulation of CD31, CD34 and CD133 expression (**Figure 6G**). Furthermore, the *in vitro* results were confirmed by qPCR assays, showing lower levels of APLN mRNA in the APLN-shRNA group compared with those in the CIA group, while levels of miR-525-5p were higher in the APLN-shRNA group than in the CIA group (**Supplementary Figure S4**). These results indicate that inhibiting APLN lowers EPC angiogenesis as well as disease activity in CIA-induced arthritis.

## Discussion

RA is well recognized for its manifestations of synovial inflammation and joint destruction (1, 37, 38). The development of RA disease relies upon pannus formation and neovascularization (2). Ang1 is a critical modulator during the physiological and pathological progression of angiogenesis (39). APLN reportedly increases IL-1β expression and VEGF-mediated angiogenesis, facilitating OA development (14, 19). Here, we report finding higher APLN and Ang1 expression in patients with RA than in those with OA. Moreover, we found that APLN stimulates Ang1 synthesis in RASFs and facilitates EPC angiogenesis by inhibiting miR-525-5p synthesis *via* PLCγ and PKCα signaling. Importantly, inhibiting the expression of APLN reduces EPC angiogenesis, reducing the progression of RA *in vivo*.

EPCs also stimulate new vessel formation (40, 41) and promotion of EPC mobilization by angiogenic factors facilitates tumor development and angiogenesis (42). EPC angiogenesis plays a vital role in RA (5, 43). EPC infiltration into joints has been reported in the CIA-induced RA mouse model (5). Here, we observed that compared with OA synovial fluid, RA synovial fluid facilitates EPC infiltration and angiogenesis, indicating that EPC-dependent angiogenesis is an important step during RA progression. Levels of EPC-specific markers were higher in our CIA mouse model than in controls. APLN shRNA reduced levels of vessel markers and EPC markers and mitigated the severity of RA disease. Thus, inhibition of APLN shows promise as a novel strategy in RA disease, reducing EPC angiogenesis and disease development.

Various proangiogenic factors, including VEGF, fibroblast growth factor, PDGF and Ang, are involved in the angiogenic process of several different diseases, including arthritis (2). Interestingly, we found that Ang1 antibody significantly antagonized increases in EPC angiogenesis induced by RA synovial fluid, suggesting that Ang1 is a vital modulator in EPC-mediated angiogenesis during RA development. Incubation of RASFs with APLN concentration-dependently promotes Ang1 synthesis, resulting in EPC angiogenesis. Importantly, Ang1 antibody, but not VEGF antibody, abolished EPC migration and tube formation in CM from APLN-treated RASFs, indicating that Ang1 is more important than VEGF in APLN-induced angiogenesis during RA disease.

Activation of the PLC/PKC signaling cascade is essential for regulating various cellular functions, including pathogenesis of arthritic diseases (34, 44). The proliferation of synoviocytes from patients with RA has been reported to be suppressed by PLC and PKC inhibitors (44). In our previous research, we found that the PLC/PKC pathway was involved in thrombin-induced interleukin-6 synthesis in rheumatoid synovial cells (45). However, the impact of these molecules on synovium-induced angiogenesis is not clear. In OA-related research, APLN increased angiogenesis responses, including endothelial cell migration, proliferation, and the capillary tube-like structure formation of endothelial cells (19, 46). Our investigations found that PLC and PKC inhibitors reduced APLN-enhanced Ang1 expression in RASFs and EPC angiogenesis. This was confirmed by findings from genetic siRNA experiments demonstrating that PLCγ and PKCα mediate the angiogenic effects of APLN. Treatment of RASFs with APLN also augmented PLCγ and PKCα phosphorylation. This suggests that PLCγ and PKCα activation are controlled by APLN-dependent Ang1 angiogenesis in EPCs. Recent publications have described how PKCδ activation regulates lymphangiogenesis and angiogenesis in RASFs and LEC cells (31, 47). However, our study showed that neither the PKCδ inhibitor (Rottlerin) nor the genetic siRNA affected APLN-facilitated expression of Ang1 and angiogenesis in EPCs, indicating that the PKCδ pathway is not involved in APLN-regulated angiogenic effects. Thus, PKCα but not PKCδ activation regulates APLN-induced Ang1 expression and EPC angiogenesis.

MiRNAs post-transcriptionally regulate gene expression (48). In RA, aberrant miRNA expression regulates the expression of inflammatory pathways (35, 36). Numerous miRNAs also control angiogenesis during RA progression (49). MiR-525-5p has been implicated in multiple cancers, including for instance glioma (50), cervical cancer (51) and NSCLC (52), but no evidence to date has indicated the involvement of miR-525-5p with RA progression. In this study, stimulation of RASFs with APLN inhibited miR-525-5p expression and transfecting them with miR-525-5p mimic antagonized APLN-promoted upregulation of Ang1 expression and EPC angiogenesis. It has been reported that transcriptional and post-transcriptional regulation play key roles in miRNA activation and inhibition (53). In this study, treating RASFs with PLCγ and PKCα inhibitors reversed APLN-promoted inhibition of miR-525-5p expression, which suggests that APLN may assist with Ang1 production and EPC angiogenesis by inhibiting miR-525-5p synthesis *via* the PLCγ and PKCα signaling cascades. Whether PLCγ/PKCα signaling regulates miR-525-5p expression through transcriptional or post-transcriptional regulation needs further examination.

**Figure 7:**
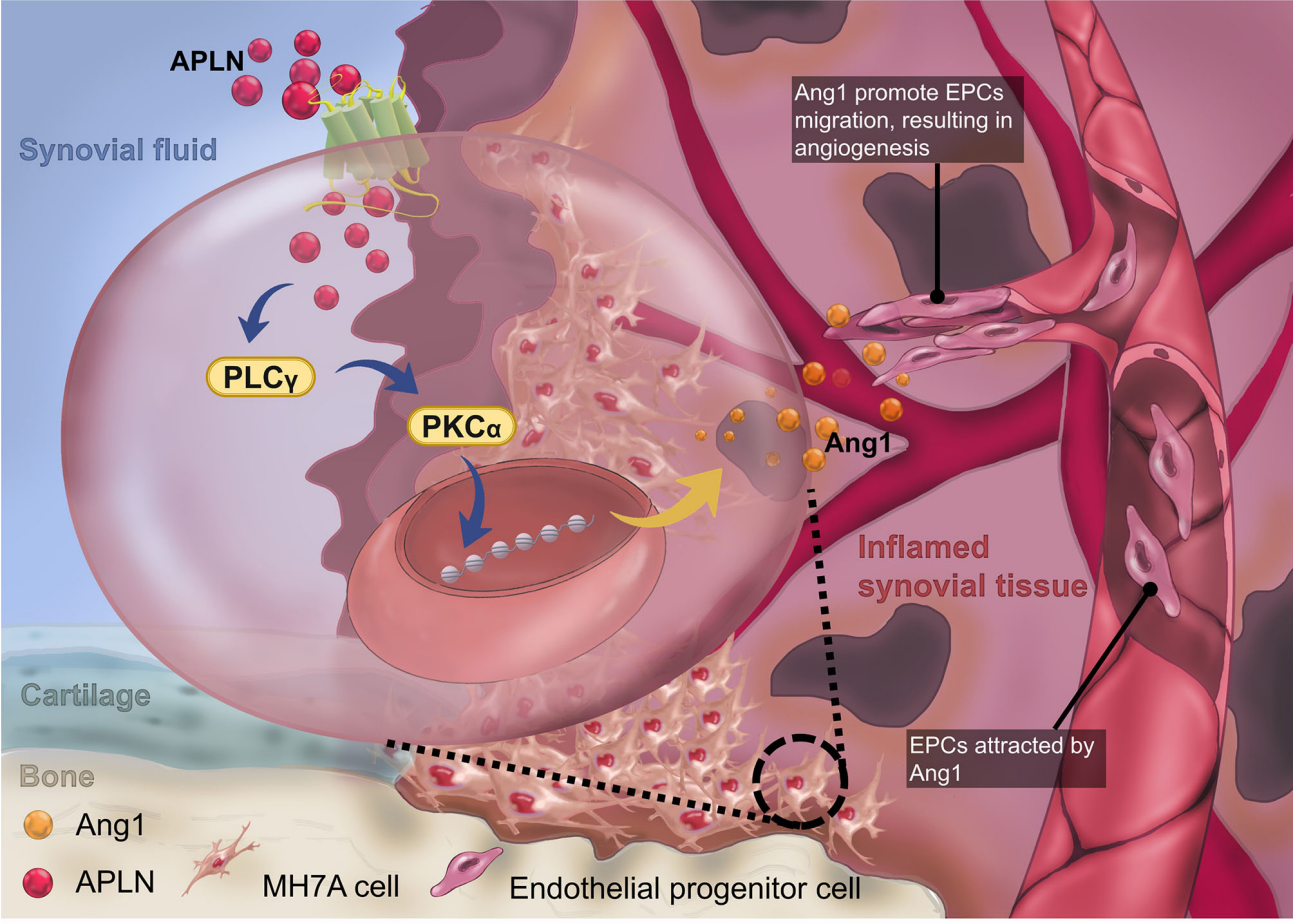
The schematic diagram summarizes the mechanisms of APLN-induced Ang1-dependent EPC angiogenesis during RA pathogenesis. APLN induces Ang1 expression in RASFs by suppressing miR-525-5p expression *via* the PLCγ and PKCα signaling pathways, and promotes EPC angiogenesis in RA.

In conclusion, we have determined that APLN increases Ang1 synthesis and subsequently facilitates EPC angiogenesis by suppressing miR-525-5p synthesis *via* PLCγ and PKCα signaling (**Figure 7**). The evidence supports the targeting of the APLN-dependent miR-525-5p/Ang1 axis in RA treatment regimens.

## Data Availability Statement 

The original contributions presented in the study are included in the article/**Supplementary Material**. Further inquiries can be directed to the corresponding author.

## Ethics Statement

The studies involving human participants were reviewed and approved by CMUH108-REC3-039. The patients/participants provided their written informed consent to participate in this study. The animal study was reviewed and approved by CMUIACUC-2019-330.

## Author Contributions

C-HTa, H-PL, and C-JH initiated the research project. Y-HZ, S-CL, C-CH, C-HTs, and S-WW performed the research. C-CH, C-HTs, and S-WW provided the material. C-HTa wrote the paper. All authors contributed to the article and approved the submitted version.

## Funding

This work was supported by grants from the Ministry of Science and Technology in Taiwan (MOST 110-2320-B-039-022-MY3; MOST 110-2314-B-039-008; MOST 110-2314-B-039-012; MOST 110-2314-B-195-003), China Medical University Beigang Hospital (110CMUBHR-07 and 110CMUBHR-11) and China Medical University Hospital (DMR-110-074).

## Conflict of Interest

The authors declare that the research was conducted in the absence of any commercial or financial relationships that could be construed as a potential conflict of interest.

## Publisher’s Note

All claims expressed in this article are solely those of the authors and do not necessarily represent those of their affiliated organizations, or those of the publisher, the editors and the reviewers. Any product that may be evaluated in this article, or claim that may be made by its manufacturer, is not guaranteed or endorsed by the publisher.
