# Supplementary material for: Apelin Promotes Endothelial Progenitor Cell Angiogenesis in Rheumatoid Arthritis Disease via the miR-525-5p/Angiopoietin-1 Pathway
Source: Front Immunol. 2021 Sep 29;12:737990. doi: 10.3389/fimmu.2021.737990 (PMC8511637; doi:10.3389/fimmu.2021.737990)
Supplement: Supplementary file 1 [file Table_1.docx]

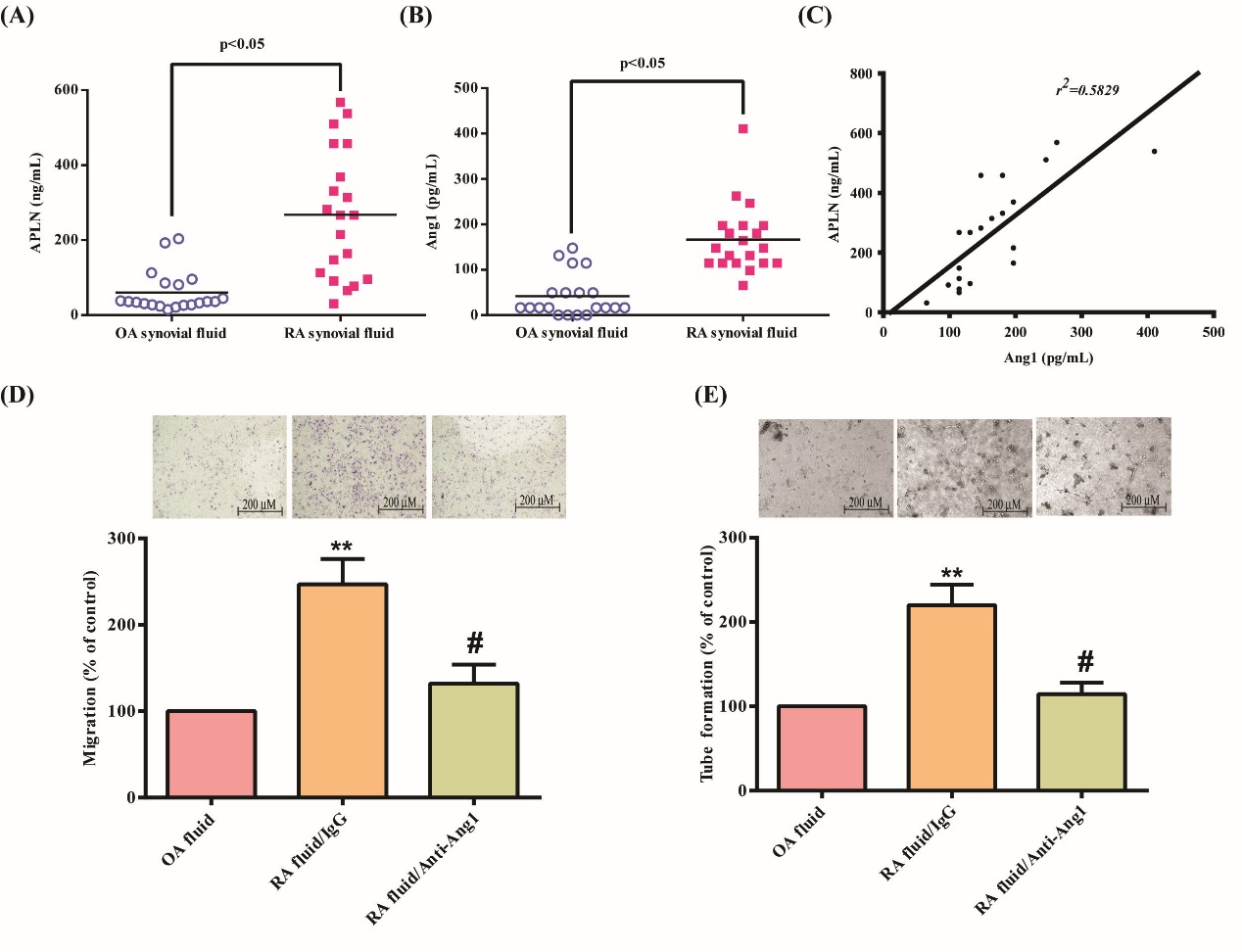


**Fig. S1. Upregulation of APLN and Ang1 expression in RA promotes EPC angiogenesis.** (A&B) APLN and Ang1 levels in OA and RA synovial fluid samples were quantified by the ELISA assay. (C) A positive correlation between APLN and Ang1 expression in RA synovial fluid samples. (D&E) RA synovial fluid was treated with or without Ang1 antibody, then applied to EPCs, before the measurement of EPC migration and tube formation. * *p* < 0.05 and ** *p* < 0.01 versus OA synovial fluid; # *p* < 0.05 versus the RA synovial fluid


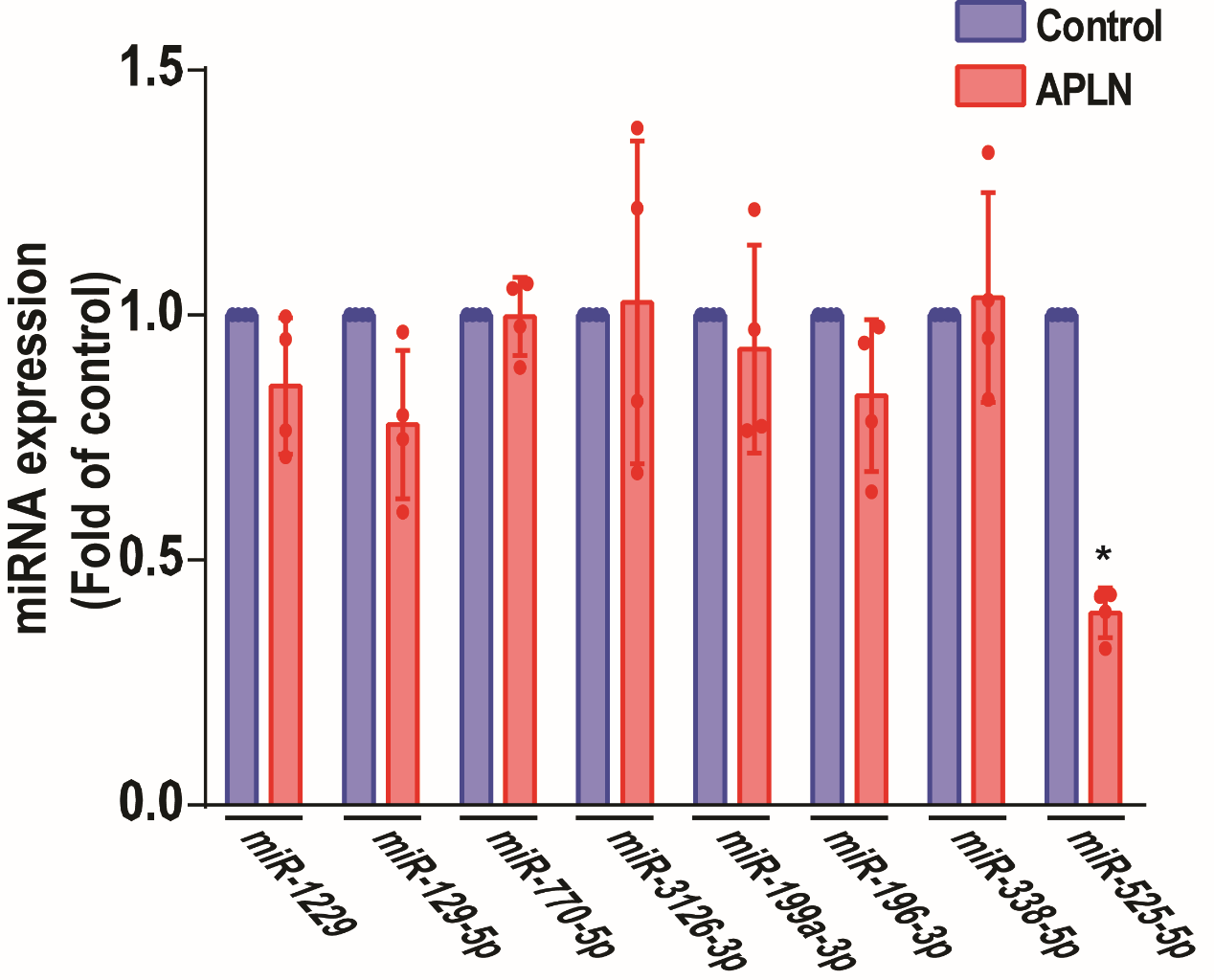


**Fig. S2**. **The APLN inhibits miR-525-5p expression.** RASFs were incubated with APLN (10 ng/ml) for 24 h. The miRNA expression was examined by qPCR. Results are expressed as the mean ± S.D. **p* < 0.05 as compared with the control group.


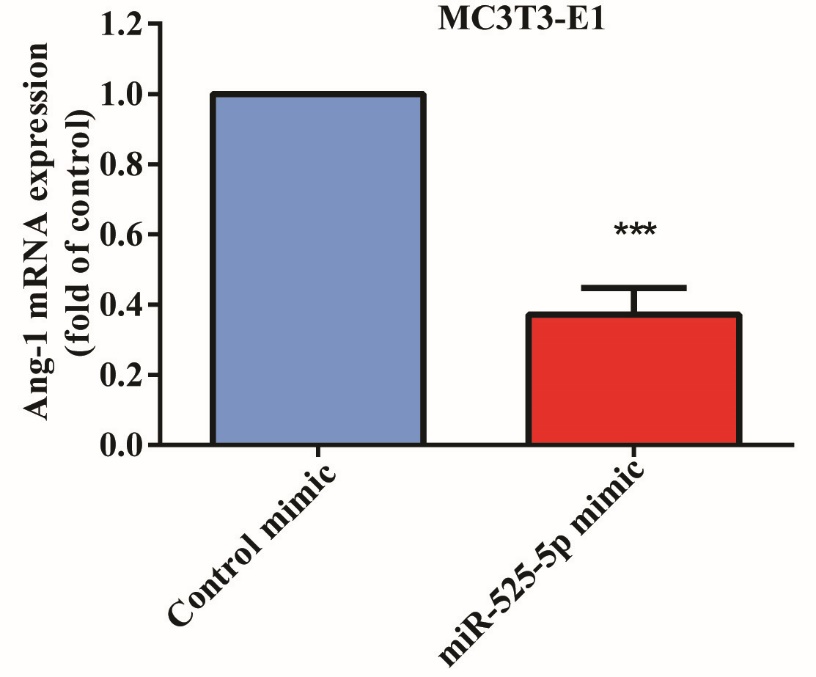


**Fig. S3**. **MiR-525-5p inhibited the Ang1 synthesis in mouse MC3T3-E1 cells.** MC3T3-E1 cells were transfected with control or miR-525-5p mimic 24 h. Ang1 levels were determined by qPCR. Results are expressed as the mean ± S.D. *** *p* < 0.001 as compared with the control group.


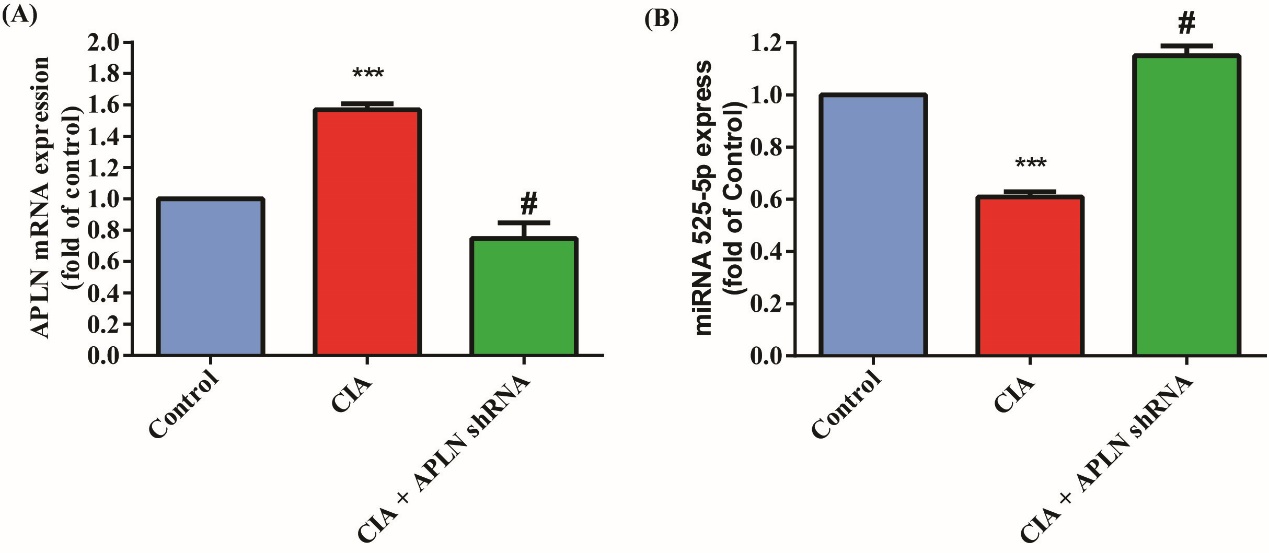


**Fig. S4**. **APLN knockdown reduces APLN and enhances miR-525-5p expression.** CIA animal model paw tissue were collected and mRNA were extracted. The APLN (A) and miR-525-5p (B) were determined by qPCR. Results are expressed as the mean ± S.D. *** *p* < 0.001 as compared with the control group. # *p* < 0.05 versus the CIA group.
